# Highly sensitive refractive index sensor based on asymmetric spectral response of TiO_2_ high-contrast grating under LED illumination

**DOI:** 10.1371/journal.pone.0354185

**Published:** 2026-07-21

**Authors:** Yua Okano, Yuusuke Takashima, Masanobu Haraguchi, Yoshiki Naoi

**Affiliations:** 1 Graduate School of Advanced Technology and Science, Tokushima University, Tokushima, Japan; 2 Institute of Post-LED Photonics, Tokushima University, Tokushima, Japan; Beni-Suef University, EGYPT

## Abstract

A highly sensitive intensity-interrogation refractive index (RI) sensor with a light-emitting diode (LED) was experimentally implemented via spectrally asymmetric resonance of a high-contrast grating (HCG). Our novel design concept realizes high sensitivity to RI variations by leveraging the asymmetry of the HCG’s resonant spectrum to control its overlap with the light-source spectrum; in contrast, most conventional designs only focus on shifting the resonant spectrum. Electromagnetic field numerical simulations clarified that an optimized HCG exhibited spectrally asymmetric resonant reflection. The measured reflection spectra showed good agreement with the calculation results, and the reflected light intensity from the sample varied dramatically with a minuscule RI variation under 490 nm LED illumination owing to a significant change in the overlap. An RI sensitivity of 481% per refractive index unit (RIU) and an RI limit of detection of 9.98 × 10^−4^ RIU were experimentally achieved. Our sensor can be used in a wide range of applications owing to its high RI sensitivity and simple LED-based system without complex equipment, such as a spectrometer or angle-resolved setup.

## Introduction

Optical sensing of the refractive index (RI) is crucial in a variety of applications, such as biosensing [1–9], gas sensing [10–12], and environmental monitoring [13,14]. Conventional optical RI sensing methods that rely on the chromatic dispersion of materials suffer from a tradeoff between sensitivity and device compactness because high-sensitivity measurements require a long optical path.

To overcome this limitation, optical resonance RI sensors have emerged as a promising technique. The resonance enhances the light–matter interaction at a certain wavelength, which leads to resonant features in the transmission, reflection, and absorption spectra. The resonant conditions are strongly influenced by the minimal RI change in the surrounding medium. Various optical resonant RI sensors using surface plasmon resonance (SPR) [15–19], localized surface plasmon resonance (LSPR) [20,21], Tamm plasmon resonance (TPR) [22–25], guided mode resonance (GMR) [26–29], high-contrast gratings (HCG) [30–36], and Fano resonance [37–40] have been reported.

Optical resonant RI sensors are primarily classified into three categories based on sensing methodologies: wavelength, angular, and intensity interrogation. In particular, an intensity-type RI sensor that measures the light intensity at the resonant wavelength has advantages for real-time sensing without angle- and wavelength-resolved measurements [41]. Several groups have reported intensity-type RI sensing systems with light-emitting diodes (LEDs) because LEDs are highly stable against environmental factors such as temperature [42]. In these approaches, the light intensity in the photodetector is dominated by the overlap between the light source and resonant spectra. Most sensor designs aim to improve the resonant spectral response to RI changes by enhancing light–matter interactions. This goal is typically achieved by concentrating the electric field on the target analyte or by increasing the overlap between the evanescent field and the analyte. [41–44]. Although the conventional sensors reported highly sensitive RI detection, further sensitivity improvements require controlling the shape of a resonant spectrum to maximize variations in the spectral overlap between the source and the resonance.

To address the above challenge, in this study, we demonstrated high RI sensitivity by utilizing the asymmetry of the resonant spectrum of an HCG to control its overlap with the light source spectrum; in contrast, most conventional designs only focus on shifting the resonant spectrum.

### Design

**Fig 1:**
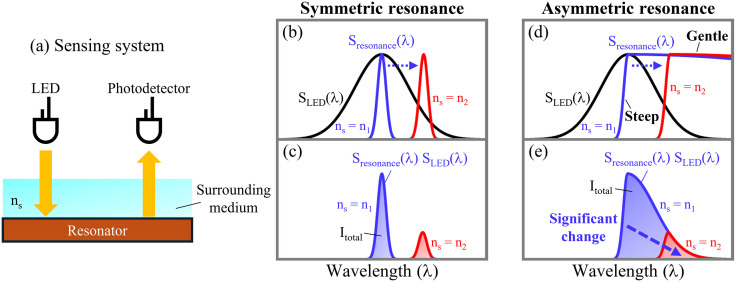
Working principle of the RI sensor. (a) Schematic of the sensing system, consisting of an LED, a photodetector, and a resonator. The RI of the medium surrounding the resonator is n_s_. (b) LED spectrum (S_LED_(λ)) and symmetric resonant spectrum (S_resonance_(λ)) with n_s_ = n_1_ and n_2_. (c) Total intensity change with RI variation in the symmetric spectrum. The curves and the colored region represent S_resonance_(λ) S_LED_(λ) and I_total_, respectively. (d) LED spectrum (S_LED_(λ)) and asymmetric resonant spectrum (S_resonance_(λ)) with n_s_ = n_1_ and n_2_. (e) Total intensity change with RI variation in the asymmetric case. The curves and the colored region represent S_resonance_(λ) S_LED_(λ) and I_total_, respectively.


The concept of highly sensitive RI sensing achieved by controlling the resonant spectrum shape is illustrated in Fig 1. The sensing system is composed of an optical resonator, LED light source, and photodetector, as illustrated in Fig 1 (a). The surrounding RI value is denoted as n_s_. The resonator is illuminated by an LED light source, and the reflected light intensity is measured using a photodiode. The total intensity entering the detector (I_total_) is determined as the integral of the product of the LED spectrum (S_LED_(λ)) and the reflection spectrum of the optical resonator (S_resonance_(λ)), where λ is the wavelength of light in vacuum:

$$\begin{array}{c} {\text{I}}_{\text{total}}=\int {\text{S}}_{\text{resonance}}(\lambda )\;{\text{S}}_{\text{LED}}(\lambda )\text{ d}\lambda \end{array}$$ (1)

When the resonant wavelength is shifted by changing n_s_, I_total_ is also varied.

To estimate the effect of the resonant spectral shape, we compared RI sensing using a typical symmetric spectrum such as GMR (Fig 1 (b) and (c)) and an asymmetric spectrum such as Fano resonance (as described in detail later) (Fig 1 (d) and (e)). When the resonant wavelength is shifted owing to a change in n_s_ (from n_s_ = n_1_ to n_2_), the shift modifies I_total_. In the case of the symmetric resonant spectrum, the spectral sharpness of S_resonance_(λ) is key to achieving high sensitivity. However, a symmetric resonant spectrum that is too sharp compared with the LED emission spectrum narrows the overlap region between S_LED_(λ) and S_resonance_(λ). This decreases the light intensity in the detector and limits the I_total_ change due to n_s_ variation, as illustrated in Fig 1 (c).

In contrast, an asymmetric resonant spectrum has the potential to improve RI sensitivity. The asymmetric resonant spectrum exhibits both steep and gentle spectral features, as shown in Fig 1 (d). The broad spectral shape on the longer-wavelength side of the resonant spectrum increases the overlap region between S_LED_(λ) and S_resonance_(λ), and the steep slope at the shorter-wavelength side of S_resonance_(λ) causes a significant change in I_total_ due to the wavelength shift of S_resonance_(λ) (Fig 1 (e)). Thus, asymmetric resonance can improve RI sensitivity.

**Fig 2:**
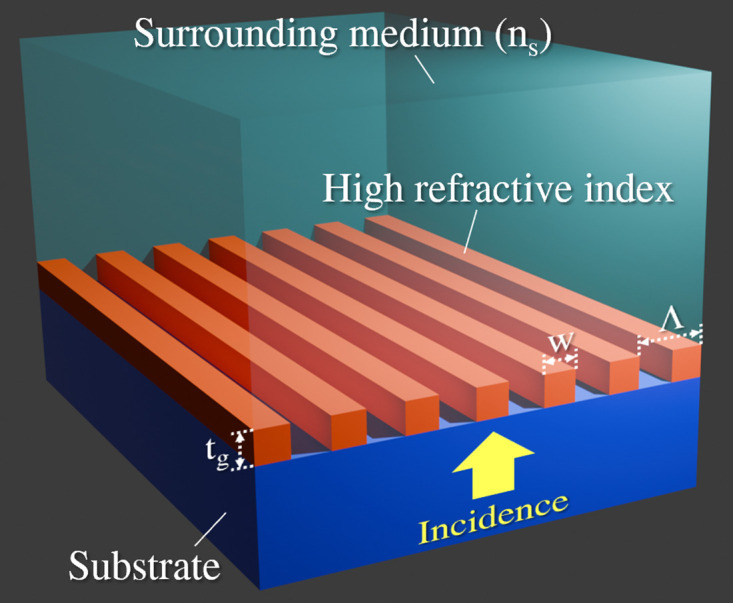
Schematic of the HCG structure. The structure is composed of a high-RI material arranged periodically on a subwavelength scale, a low-RI medium surrounding the structure, and a substrate. Light is incident on the structure from the substrate side. The symbols Λ, w, and t_g_ denote the grating period, bar width, and thickness, respectively.

Using an HCG is an attractive option for achieving an asymmetric-shape reflection spectrum. Fig 2 shows a schematic of the HCG. The HCG consists of a high-RI material arranged with subwavelength periodicity, a low-RI medium surrounding the structure, and a substrate. The symbols Λ, w, and t_g_ in Fig 2 denote the grating period, bar width, and thickness, respectively.

**Fig 3:**
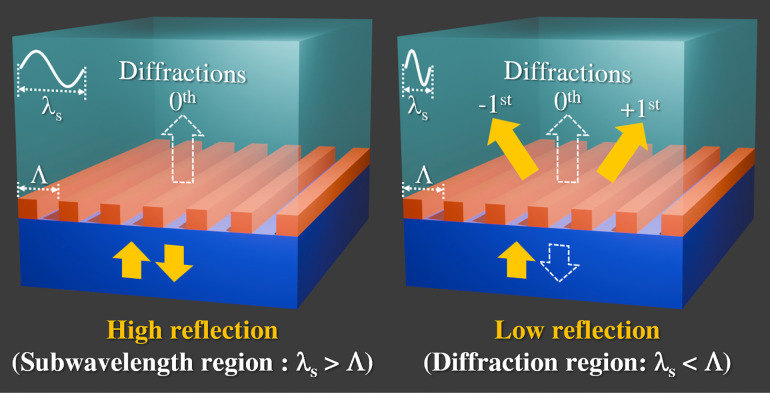
Concept of coupling between eigenmodes and diffractions. The wavelength of light in the surrounding medium is represented by λ_s_. High reflection is obtained in the resonant condition for λ_s_ > Λ (subwavelength region). The reflectivity abruptly decreases for λ_s_ < Λ (diffraction region) owing to the energy flowing through higher-order diffractions.

When light enters the HCG (from the substrate side in this study), the periodic RI distribution imparts additional in-plane momentum to the light. This leads to coupling between the incident light and optical eigenmodes originating from the RI periodicity. The excited eigenmodes inside the HCG further couple with several diffractions in the surrounding medium. The coupling efficiency between the eigenmodes and diffractions changes abruptly based on the ratio of the wavelength of light in the surrounding medium (λ_s_) to Λ [45] (Fig 3).

When λ_s_ > Λ (subwavelength region), only 0^th^ order diffraction (i.e., transmitted light) propagates the optical energy because of its subwavelength period (higher-order diffractions are evanescent waves). Owing to the electromagnetic boundary conditions, the coupling of the eigenmodes and 0^th^ order diffraction is determined by the matching between both electromagnetic field profiles at the top interface of the HCG [45]. The coupling efficiency simply depends on the spatial average of the eigenmode field at the interface, because the 0^th^ order diffracted light exhibits a spatially uniform field profile [45]. In particular, the high RI contrast of the HCG generates multiple eigenmodes inside the HCG, and the superposition of these modes facilitates the control of the field profile at the interface. The wavenumbers of the eigenmodes are given by the period and bar width because the in-plane momentum is provided by the RI periodicity. The grating thickness determines the propagation distance of the eigenmodes and thus controls its phases. Suitable selections of the HCG’s structural parameters (Λ, w, and t_g_) reduce the electromagnetic field average at the top interface, thereby suppressing the coupling of the eigenmodes and 0^th^ order diffraction. As a result, most of the incident light energy is reflected back. In particular, high reflectivity can be obtained by suppressing only the 0^th^ order diffraction because the higher-order diffractions are evanescent waves in the subwavelength region. This provides ultra-broadband high-reflection characteristics [45].

For λ_s_ < Λ (diffraction region), higher-order diffractions carry the optical energy. Energy propagation through higher-order diffractions abruptly reduces the reflectivity of the HCG. Thus, an asymmetric-shape reflection spectrum with sharp and broad spectral features can be obtained.

Based on the aforementioned concept, we design the structural parameters of HCG for an RI sensor using LED illumination. TiO_2_ is used as the HCG material [46], and sapphire is used as the substrate [47]. A transverse electric (TE) wave (the electric field oscillates along the grating bars) is normally incident on the TiO_2_–HCG structure from the sapphire side. HCG is surrounded by water (n_s_ = 1.333) because various applications of RI sensors such as biosensing are performed in aqueous environments [1]. Assuming 490 nm LED illumination, we set Λ = 350 nm, w = 125 nm, and t_g_ = 156 nm by considering the dispersion relationship of the eigenmodes inside TiO_2_–HCG [45].

**Fig 4:**
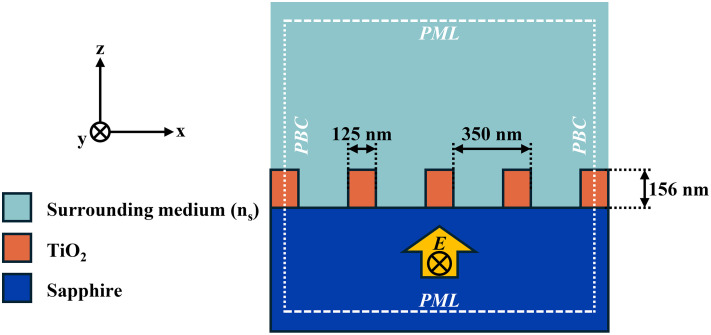
FDTD calculation model for the proposed structure. The HCG material and substrate are TiO_2_ and sapphire, respectively. The RI of the surrounding medium is n_s_. The grating period, bar width, and thickness are Λ = 350 nm, w = 125 nm, and t_g_ = 156 nm, respectively. TE-polarized light with the electric field oriented along the y-direction is normally illuminated from the substrate side. PBC and PML conditions are set as the x- and z-boundaries, and the structure extends infinitely in the y-direction.

We calculated the electromagnetic field distribution near the structure using a finite-difference time-domain (FDTD) method (Fullwave: Synopsys) to estimate the reflection characteristics. Fig 4 shows the FDTD simulation model. In this calculation, the RI chromatic dispersion of the TiO_2_ (n_TiO2_ = 2.2054 at λ = 490 nm) and sapphire (n_sapphire_ = 1.7752 at λ = 490 nm) were taken from experimental reports [46,47]. Periodic boundary conditions (PBC) and perfectly matched layer (PML) conditions were applied as the x- and z-boundaries, respectively. The structure was assumed to continue infinitely in the y-direction.

**Fig 5:**
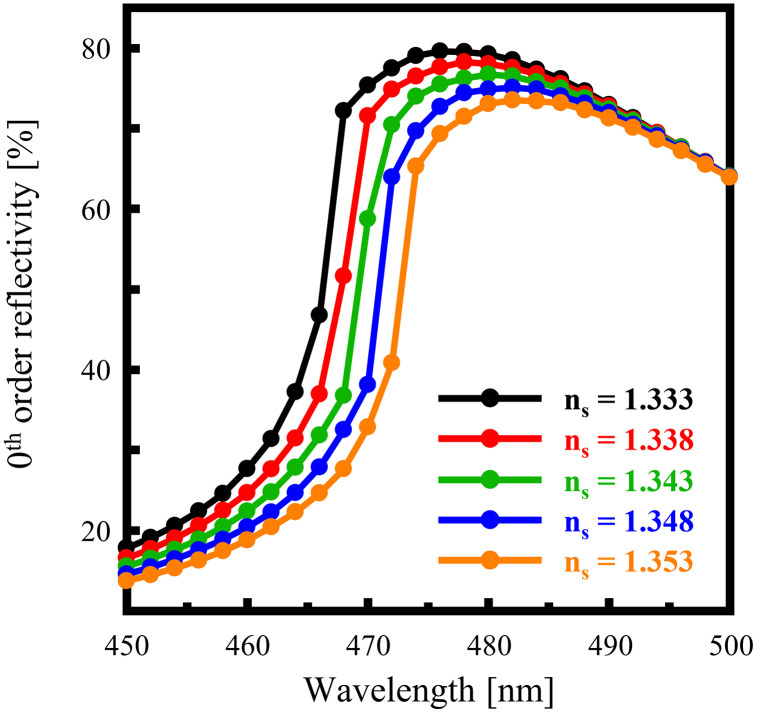
Calculated reflection spectra of the proposed structure with variations in the surrounding RI.

The calculated 0^th^ order reflectivity of TiO_2_–HCG is plotted in Fig 5. For n_s_ = 1.333, an asymmetric-shape reflection spectrum with a peak wavelength of 476 nm is observed. The gradient of the spectrum around the peak wavelength is steep on the shorter-wavelength side and gradual on the longer-wavelength side. The peak wavelength is significantly red-shifted and the peak reflectivity decreases with increasing n_s_ from 1.333 to 1.353, because the electromagnetic field profile at the top interface is modified owing to the surrounding RI change.

**Fig 6:**
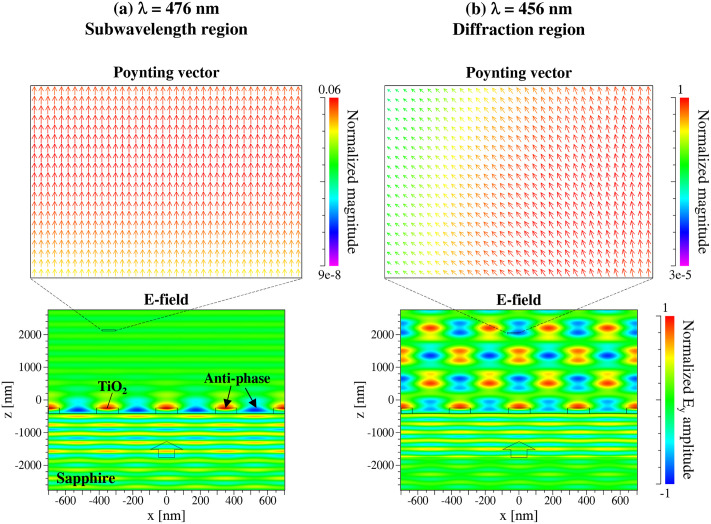
Normalized electric field (E_y_) distribution near the proposed structure and transmission-side Poynting vector distributions. (a) λ = 476 nm. (b) λ = 456 nm. λ denotes the wavelength of light.

Fig 6 shows the y-component of the electric field (E_y_) near the TiO_2_–HCG structure at (a) 476 nm and (b) 456 nm for n_s_ = 1.333. Fig 6 also shows the Poynting vector distributions of light transmitted through the HCG.

At the peak wavelength of 476 nm (Fig 6 (a)), the E_y_ field distribution of the 0^th^ order diffraction on the transmission side exhibits a uniform field profile, and the Poynting vectors point only in the z-direction. The coupling between the 0^th^ order diffraction and the eigenmodes is determined by the spatial average of the eigenmode E_y_ field at the top interface of the HCG. The E_y_ fields at the grating bars (TiO_2_) and gap of the HCG are anti-phase at the interface, and the average E_y_ field at the interface approaches zero; thus, the coupling is suppressed. Consequently, a large amount of incident energy is reflected back, and the Poynting vectors of the transmitted light show a small amplitude. At the wavelength of 456 nm (Fig 6 (b)), higher-order diffractions on the transmission side exhibit periodic oscillation along the x-direction. Because the periodic field distribution of the higher-order diffraction matches well with the eigenmode field profile at the interface, the eigenmodes can couple with higher-order diffractions. As a result, higher-order diffractions carry the optical energy, and the Poynting vectors possess both z- and x-components, as well as much larger amplitudes than those at the wavelength of 476 nm. The mode coupling causes an abrupt decrease in reflectivity. Consequently, the asymmetric resonant spectral shape is obtained.

### Experimental results and discussion

**Fig 7:**
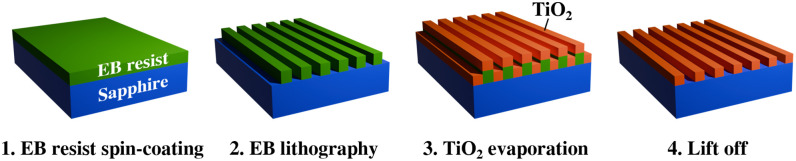
Fabrication process of the proposed structure.

**Fig 8:**
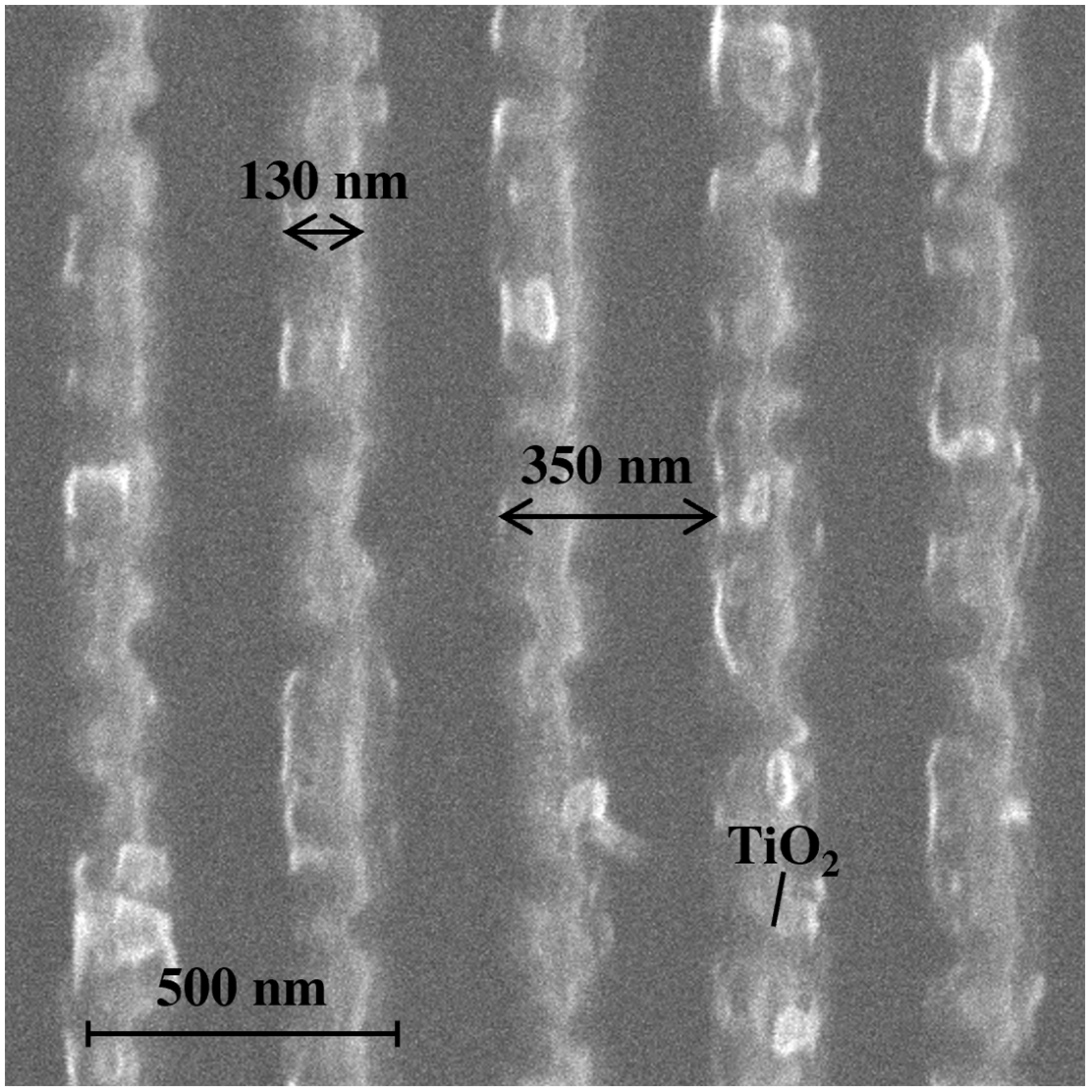
Top-view SEM image of the fabricated structure. The scale bar indicates 500 nm.

Fig 7 illustrates the lift-off process of the proposed sensor. An electron-beam resist (ZEP520A: Zeon) diluted with anisole was spin-coated onto the sapphire substrate. The resist coating was exposed to the electron beam for a 1.2 × 1.2 mm region after pre-baking the substrate (pre-baking: 120 ℃, 30 min). The sample was developed using ZED-N50 (Zeon). Subsequently, a TiO_2_ film of 156 nm thickness was deposited on the resist pattern using vacuum evaporation, and the resist was removed using N-methyl-2-pyrrolidinone. Fig 8 shows a scanning electron microscopy (SEM) image of the fabricated structure. The grating period was approximately 350 nm, as designed. We evaluated the grating bar widths at multiple locations in the SEM images of the sample. The 400-point average was 129 nm, which was generally consistent with the designed dimension of w = 125 nm.

**Fig 9:**
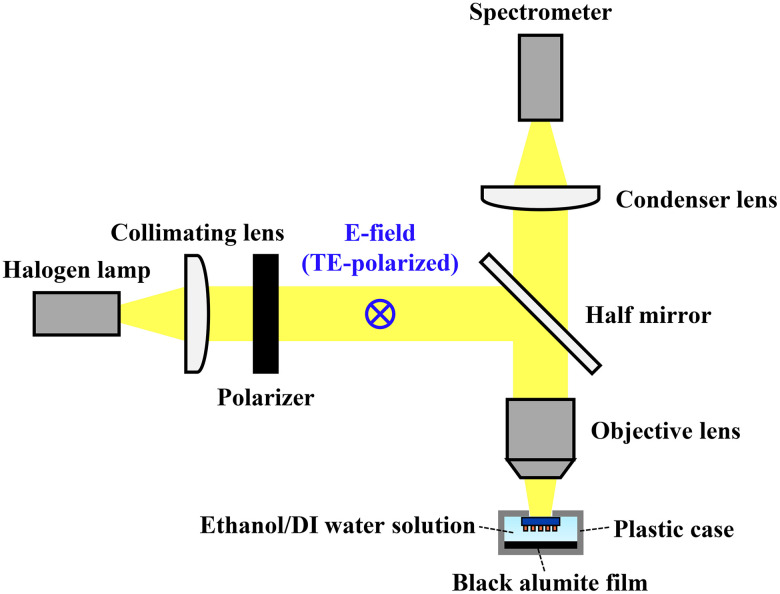
Optical system for measuring the reflection spectrum of the fabricated structure. For the reflection spectrum measurements, a halogen lamp and spectrometer were used as a light source and detector, respectively. For the RI sensing, a cyan LED and a photodetector were set instead of the halogen lamp and the spectroscope.

The reflection spectrum of the fabricated TiO_2_–HCG structure was measured in an aqueous environment using the optical system shown in Fig 9. The sample was placed in a plastic case and surrounded by an ethanol/deionized (DI) water solution to control the surrounding RI value by changing the ethanol concentration. The RI value corresponding to each ethanol concentration was measured using a refractometer (PR-RI: Atago). A black alumite film was placed in the sample case to suppress the reflection from its bottom.

A halogen lamp was employed as the broad light source to evaluate the reflected spectral shape of the sample in the visible region. TE-polarized light passed through the polarizer was focused on the TiO_2_–HCG structure from the side of the sapphire substrate using an objective lens, and the reflection spectrum was obtained using a spectrometer (OP-FLAME-S: Ocean Photonics).

**Fig 10:**
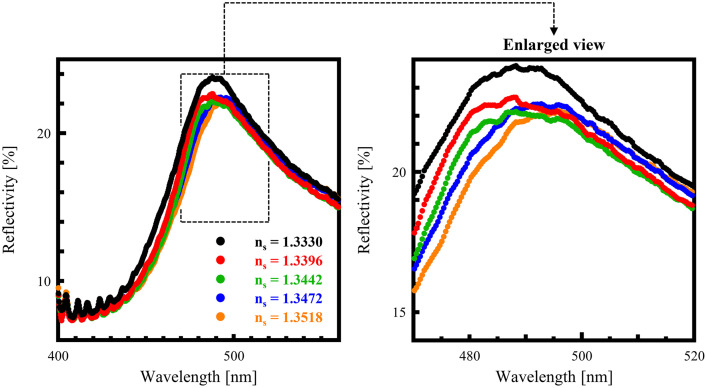
Reflection spectra of the fabricated sensor. Measured reflection spectra of the fabricated sample with variations in the surrounding RI and the enlarged view around the peaks.

Fig 10 shows the reflection spectra of the fabricated sample. The spectra were measured at different ethanol concentrations in a random order. When the sample was surrounded by DI water (n_s_ = 1.3330), an asymmetric resonance peak was obtained near 488 nm (the slope of the shorter-wavelength side was steep, while that of the longer-wavelength side was gentle). Increasing the RI of the ambient medium yielded both resonant wavelength shifts toward longer wavelengths and a decrease in peak reflectivity.

These measurement results are consistent with the FDTD estimations, despite the spatial fluctuation of the grating bar width (the standard deviation measured at 400 points was 32.7 nm) (Fig 8). To evaluate the influence of the fabrication error in the bar width, FDTD simulations were performed for grating bar widths of the designed value ±30 nm (where the variation of ±30 nm approximately corresponds to the measured standard deviation of the bar width). The peak wavelength and its shift due to RI modification were unchanged compared with those of the designed width; only the full width at half maximum slightly varied within ±4 nm. The results show that bar-width variations caused only negligible changes in the optical properties. Thus, our structure possesses high tolerance to width fluctuations; this tolerance is highly useful for practical fabrication.

**Fig 11:**
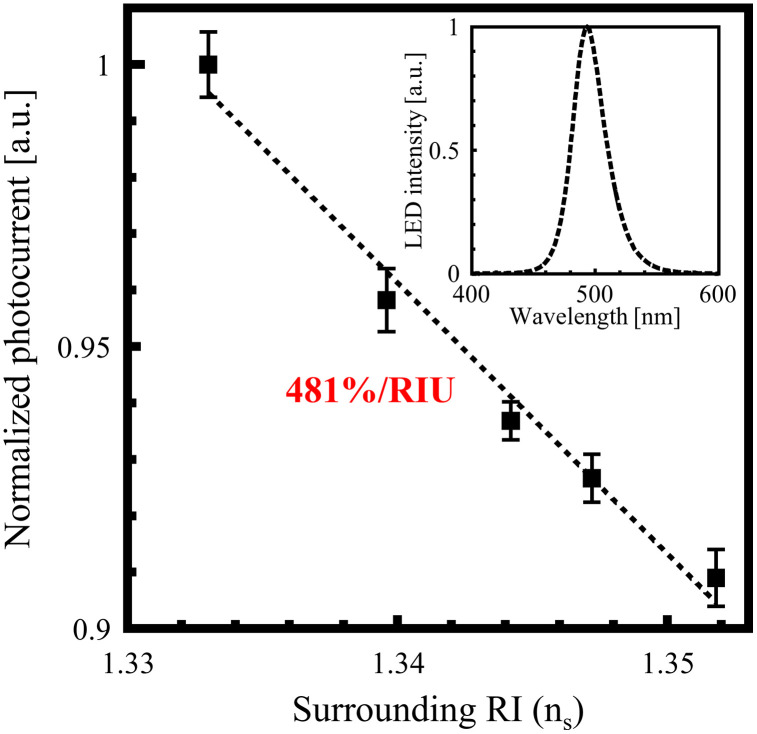
Normalized photocurrent variations to the surrounding RI change under cyan LED illumination. The error bars represent three standard deviations (±3σ). The inset shows the LED spectrum in this measurement.

The RI sensitivity of the fabricated HCG was evaluated using a cyan LED (peak wavelength: 493.5 nm, full width at half maximum: 30.8 nm) and a photodiode instead of a halogen lamp and spectroscope, as shown in Fig 9. The LED was driven at a constant current (36 mA) to minimize fluctuations in light intensity (optical power: 3 mW). The reflected light intensity was measured as the photocurrent of the photodiode using a Keithley Model 6517A electrometer/high resistance meter, and the other experimental conditions were identical to those used for the reflection spectrum measurement. Fig 11 illustrates the dependence of the reflected light intensity on the n_s_ value. The LED spectrum used in the experiment is shown in Fig 11. The filled squares show the average intensity over 1000 measurements, which decreased with an increase in the surrounding RI value. The error bars indicate three standard deviations (±3σ) and account for the effects of noise in the system, such as fluctuations in LED intensity, noise from the photodetector and electronic circuit, and environmental perturbations (e.g., ambient light). The ± 3σ error bars were also derived from 1000 measurements, indicating the high repeatability of the measurements. The linear fit in Fig 11 shows an R² value of 0.982, which suggests a good linear relationship between the surrounding RI and the reflected light intensity. The experimental relative intensity change, namely sensitivity, reached 481% per refractive index unit (RIU).

Additionally, we evaluated the limit of detection (LOD) of our RI sensor. The LOD is determined by the standard deviation (σ) during the measurement and RI sensitivity (S). We defined the LOD as 3σ/S [48]. The LOD determined in this study was as low as 9.98 × 10^−4^ RIU, using the average value of σ in the measurement range.

The RI sensitivity of the proposed structure is much higher than those of intensity-interrogation-based sensors using LEDs and photodetectors [41–44,49–51] (Table 1). This high sensitivity results in an LOD comparable to those of other sensors using special techniques for reducing signal noise [50,51].

**Table 1:** Comparison of the proposed sensor with other intensity-interrogation-based sensors operating with LEDs and photodetectors.

Sensor structure	RI sensitivity (S) [%/RIU]	LOD (3σ/S) [RIU]	Incidence
GMR [41]	99.9	1.23 × 10^−4^	Oblique
GMR [42]	18.6	9.21 × 10^−5^	Oblique
Waveguide-based LSPR [43]	36	4.29 × 10^−4^	Facet coupling
GMR [44]	39	2.45 × 10^−4^	Normal
Photonic crystal [49]	~159	—	Oblique
Waveguide [50]	30.242	1.70 × 10^−3^	Facet coupling
Bent waveguide [51]	39.2	1.59 × 10^−3^	Facet coupling
This study	481	9.98 × 10^−4^	Normal

This sensor is applicable to a variety of applications owing to its high RI sensitivity and simple measurement configuration without complex and bulky equipment, such as a spectrometer and an angle-resolved system.

## Conclusion

A highly sensitive RI sensor with an LED light source was designed using the asymmetric-shape resonant reflection spectrum of TiO_2_–HCG. The spectral asymmetry realized a high RI sensitivity under 490 nm LED illumination. The experimental RI sensitivity reached 481%/RIU and corresponded to an RI LOD of 9.98 × 10^−4^ RIU. Our sensor can be applied in various fields owing to its high RI sensitivity and simple, low-cost LED-based measurement system without a complex and bulky spectrometer or angle-resolved setup.
